# Flux equipartition in astrophysical plasma turbulence

**DOI:** 10.1126/sciadv.adv8988

**Published:** 2025-07-02

**Authors:** Raffaele Marino, Jin-Han Xie

**Affiliations:** ^1^ CNRS, École Centrale de Lyon, INSA Lyon, Université Claude Bernard Lyon 1, Laboratoire de Mécanique des Fluides et d’Acoustique, UMR5509, F-69134 Écully, France.; ^2^ Department of Mechanics and Engineering Science at College of Engineering and State Key Laboratory for Turbulence and Complex Systems, Peking University, Beijing 100871, PR China.

## Abstract

Expanding plasmas are ubiquitous in the Universe, from supernovae to stellar atmospheres and winds, carrying various forms of energy. Crucial for understanding their behavior, the characterization of the scale-to-scale energy transfer resulting from the interplay of turbulent motions, propagating waves, and instabilities is a key scope of major space missions. Here, we show how simultaneous upscale and downscale energy transfers occur in solar wind, leading statistically to equipartition of the turbulent energy flux. Our study sheds light on the paradigm of the existence of dual energy cascades in astrophysical plasmas, identifying the scales at which energy sources act in the magnetohydrodynamic regime driving turbulent dynamics in solar wind. These findings suggest that a significant fraction of the energy injected into stellar winds at scales much smaller than those of galaxies could be transferred to larger scales through turbulence, potentially influencing star formation processes.

## Teaser

Dual energy cascades occur in astrophysical plasmas leading statistically to equipartition of the turbulent flux in stellar winds.

## INTRODUCTION

Astrophysical plasmas comprise 99% of the visible Universe, pervading the interstellar space unhindered for billions of kilometers or being shaped by gravity and magnetic fields in the proximity of planets and within the stars (*1*). They indeed represent the main constituent of an incredibly large number of celestial bodies and frameworks in outer space—including solar wind, a supersonic and super-Alfvénic plasma flow composed of ionized hydrogen (protons and electrons), alpha particles, and a small fraction of heavier ions—that originates from the solar corona and expands far past the orbits of the solar system planets, filling the interplanetary space (*2*). Being weakly collisional, carrying the Sun’s magnetic field, solar wind supports the propagation of multiple types of plasma waves and is highly turbulent (*3*, *4*). The interstellar medium is mainly composed of gas, predominantly in the form of ionized, atomic, and molecular hydrogen. Observations show a close correlation between the star formation rate and the surface density of the galactic gas, known as the Schmidt-Kennicutt relation (*5*). The star formation rate is typically about two orders of magnitude lower than the simplest theoretical estimate obtained by dividing the mass of dense gas by the system’s free-fall time, which is interpreted as evidence for the presence of physical processes that oppose gravitational collapse. Two primary mechanisms have been proposed to explain this discrepancy: the influence of magnetic fields (*6*) and the effect of turbulence (*7*). Theoretical and numerical studies suggest that the turbulence observed in molecular clouds (*8*) interacts with gravity in distinct ways. It can broaden the density probability distribution function of the interstellar medium, leading to the formation of high-density regions that are more likely to become gravitationally unstable (*9*), thereby promoting star formation. At the same time, turbulence provides support against gravitational collapse through turbulent velocity dispersion, which in turn reduces the star formation rate (*10*). These effects are central to the theory of turbulent fragmentation (*11*, *12*); however, it remains unclear what exactly drives turbulence in molecular clouds. In present-day spiral galaxies, such as the Milky Way, there is numerical evidence that stellar feedback—resulting from a combination of ionizing radiation, stellar winds, and supernovae—is the dominant mechanism driving turbulence (*13*, *14*). On the other hand, in galaxies with higher gas densities, large-scale processes may play a prominent role in driving turbulence (*15*). Simulations that incorporate both large-scale turbulent driving and stellar feedback suggest that models relying solely on the latter may fail to accurately reproduce the Schmidt-Kennicutt relation (*10*).

Within the heliosphere, at time and length scales comparable to that of dynamics and structures developing on the Sun, solar wind turbulence is well described in the frame of magnetohydrodynamics. At sub-ion scales, the kinetic effects dominate, and the fate of the energy stored in space plasmas is determined by these phenomena as magnetic reconnection (*16*), particle acceleration, and wave-particle resonances (*17*), leading to dissipation and local heating of the interplanetary medium (*18*). Characterizing the scale-to-scale transfer and the direction (sign) of the turbulent energy flux in solar wind and identifying dominant energy injection scales, phenomena, and regimes at which dissipation takes place are major space physics puzzles. In 1968 Coleman first suggested that the heliospheric plasma could be in a turbulent state, showing that (at magnetohydrodynamic scales) the solar wind magnetic energy spectrum is characterized by a power-law scaling reminiscent of the inertial range typical of hydrodynamic turbulence (*19*). Belcher and Davis (*20*) then observed that large portions of the solar wind are in the so-called Alfvénic state, characterized by velocity and magnetic field fluctuations having a high degree of correlation. This is associated to weakening nonlinear couplings between plasma modes, as it stems from the magnetohydrodynamic equations arranged in terms of the Elsässer variables (*21*)

$$\begin{array}{l} \frac{\partial }{\partial_{t}}z^{\pm }+\left(z^{\mp }\cdot \nabla \right)z^{\pm }=\\ \left(\frac{\nu \pm \lambda }{2}\right)\nabla^{2}z^{\pm }+\left(\frac{\nu \mp \lambda }{2}\right)\nabla^{2}z^{\mp }-\frac{1}{\rho }\nabla P_{\text{tot}} \end{array}$$ (1)

The fields $z^{\pm }$ combine the proton velocity ( v ) and the magnetic field in Alfvén units ( $b=B/\sqrt{4{\pi \rho }_{0}}$ , where $\rho_{0}$ is the mean plasma density). When the background magnetic field points toward the Sun the Elsässer variables are typically defined as $z^{\pm }=v\pm b$ ( $z^{\pm }=v\mp b$ if the polarity is the opposite), so that the $z^{+}$ fluctuations correspond to Alfvénic modes propagating outward with respect to the Sun, while the $z^{-}$ is inward-traveling Alfvénic modes in the solar wind frame (*22*). In Eq. 1, $P_{\text{tot}}$ is the total pressure, whereas ν and λ are the kinematic viscosity and magnetic resistivity, respectively. Turbulent dynamics are suppressed when the plasma is Alfvénic, being either $z^{-}≃0$ or $z^{+}≃0$ , which prevents the energy to be efficiently transferred by the nonlinear terms in Eq. 1. The observation of extended power law spectral ranges in the Alfvénic solar wind is a state known as Alfvénic turbulence (*23*). Since the early solar wind missions, numerous studies have shown that there is an imbalance between the energy per unit of mass associated with $z^{+}$ and $z^{-}$ fluctuations, respectively, $e^{+}={|z^{+}\;|}^{2}$ and $e^{-}={|z^{-}\;|}^{2}$ (*2*). The Elsässer ratio $r_{e}=e^{-}/e^{+}$ is indeed observed to be significantly smaller than 1 close to the Sun, increasing to ~0.5 at ~2.5 astronomical units (au), then leveling off around this value at greater distances (*24*). The heliocentric distance at which the solar wind speed exceeds the phase speed of Alfvénic and fast-mode magnetohydrodynamic waves identifies the Alfvénic critical point, beyond which only outward-propagating Alfvén waves of solar origin can be detected. Being faster than the solar wind bulk speed, inward Alfvén waves generated before this point do precipitate back to the Sun. As a consequence, the inward-propagating modes observed beyond the critical point cannot have a solar origin and must have been created locally, whereas outward-propagating modes can have both solar and local origins. Several mechanisms for the local generation of the minority sunward component of the fluctuations have been proposed, which would act to compensate the imbalance and support the nonlinear mode couplings as the plasma expands, among which the parametric decay instability (*25*, *26*) and solar wind shear (*27*, *28*).

The presence of a fully developed turbulent state in neutral fluids can be rigorously assessed by verifying the validity of the Kolmogorov 4/5 law that connects the third-order longitudinal structure function of the velocity and the mean rate at which kinetic energy is transferred across the inertial range (*29*). Assuming incompressibility, homogeneity, and local isotropy, a proportionality relation between the mixed third-order moment of the longitudinal increments of the Elsässer variables [ $\Delta z^{\pm }\left(r\right)$ ] and the increment scale (*r*), analogous of the 4/5 law, was proposed for the inertial range in magnetohydrodynamics by Politano & Pouquet (P&P) (*30*, *31*): $\langle {|\Delta z^{\pm }\left(r\right)|}^{2}\Delta z_{\|}^{\mp }\left(r\right)\rangle =-\frac{4}{3}\epsilon^{\pm }r$ , where $\epsilon^{\pm }$ are the mean pseudo-energy transfer rates. The validity of the P&P law in solar wind was proven in a large number of studies (*28*, *32*–*36*), demonstrating the existence of well-defined inertial energy cascades in heliospheric plasmas, which helped address the conundrum on the existence of Alfvénic turbulence in the outer space. Surrogates of the original P&P law have been proposed over the years to soften its hypotheses and in many cases tested against observations (*37*). Estimates of the incompressible turbulent energy transfer rate in the interplanetary plasma, $\epsilon_{I}=\left(\epsilon^{+}+\epsilon^{-}\right)/2$ , have thus been obtained, making it possible to assess the contribution of the turbulent cascade to solar wind heating (*28*, *38*). The fact that solar wind expands may ultimately contribute to reducing its plasma energy density as it propagates throughout the heliosphere, the primary magnetohydrodynamic balance being indeed between the energy increase due to advective flux and the reduction of energy through expansion and dissipation (*39*). On the other hand, studies of both Alfvénic and non-Alfvénic solar wind streams have shown that expansion may have an appreciable damping effect on $e^{+}$ and $e^{-}$ only at large scales ( ≳ hours), while it is negligible at smaller scales where the turbulent cascade dominates (*40*). It has long been debated in the literature whether the assumptions of incompressibility and local isotropy, underlying the P&P law, would result in over- or underestimations of the cascade rate in solar wind. Although the magnetohydrodynamic inertial range of solar wind turbulence encompasses both incompressible and compressible fluctuations, only ~5 to ~20% (depending on the distance from the Sun) of the solar wind energy is due to the compressible component (*41*–*43*), the Alfvén waves being the dominant (incompressible) component. Early attempts to incorporate compressibility into the original P&P law were based on heuristic arguments (*44*, *45*), revealing a substantial increase of the estimated compressible cascade rate compared to the incompressible cascade rate in solar wind. Analytical approaches to derive a compressible third-order law were developed thereafter and applied to a wide range of datasets (*43*, *46*, *47*). These formulations have generally shown that the compressible energy transfer rate, $\epsilon_{C}$ , remains comparable to the incompressible energy transfer rate, $\epsilon_{I}$ , with a marked increase in the ratio $\epsilon_{C}/\epsilon_{I}$ observed in a relatively small fraction of intervals [~10% of the cases reported in (*46*)]. Moreover, density fluctuations were found to affect the energy cascade rate in the slow solar wind more than in the fast solar wind. The presence of the interplanetary magnetic field induces various types of anisotropy (*48*). Some studies have compared the solar wind turbulent cascade rates derived from the P&P law with those obtained from alternative third-order formulations that incorporate anisotropic effects and have found overall agreement between the two approaches (*35*). On the other hand, observational and numerical investigations have also shown how the incompressible turbulent energy flux developing in the parallel direction—along the background magnetic field—can in certain conditions be consistently smaller than the perpendicular energy flux, in both fast and slow solar wind streams (*33*, *49*, *50*). Last, estimates based on observations from the Parker Solar Probe (*51*) have revealed that the incompressible energy transfer rate ( $\epsilon_{I}$ ) increases significantly at distances close to the Sun, with values at 0.17 au being ~100 times higher than those at 1 au (*36*, *50*). Although switches in the sign of $\epsilon_{I},\epsilon^{\pm },\epsilon_{C}$ obtained from solar wind measurements were reported in the literature and often interpreted as an indication of inverse energy cascades (*32*, *43*, *45*, *52*), the lack of identification of the scales at which energy is primarily injected into the expanding solar wind, possibly triggering an upscale flux, together with the difficulty in computing the scale-to-scale turbulent energy transfer from observations, has prevented the community from drawing conclusions about the existence of dual energy cascades in astrophysical plasmas.

Here, we provide evidence of the occurrence of dual energy cascades in solar wind, identifying the dominant scales at which the interplanetary plasma is forced in the magnetohydrodynamic regime. To prove this paradigm, a forcing-scale resolving third-order statistical approach, alternative to traditional scaling laws (*37*), is implemented to analyze the high-latitude fast solar wind observed by the spacecraft Ulysses (*53*). We present estimates of the local-in-scale total energy flux, showing the onset of bidirectional energy transfers and how they can lead statistically to the equipartition of the turbulent flux in expanding astrophysical plasmas. These results provide insights into the origin of turbulence in interplanetary space and solar wind energetics. Moreover, they suggest that stellar winds, by sustaining the transfer of energy to scales larger than those of individual stars, may act as a source of turbulence, driven by processes occurring at scales far smaller than those of galaxies.

## RESULTS

### Dual energy cascade in solar wind

Unlike the mean pseudo-energy transfer rate ( $\epsilon^{\pm }$ ) in the P&P law—a quantity that does not depend on the scale, representing an average over the inertial range—our approach is able to provide the scale-dependent total energy flux *F*(*r*) from observational data (*54*), even in the case that energy is injected within the range of scales analyzed. Being an analog of the cumulative transfer function built on the nonlinear terms of Eq. 1 (*55*), the function *F*(*r*) allows us to assess the sign (direction) of the turbulent energy transfer at each accessible scale *r* in the space plasma under study. Here, we analyze fast solar wind samples collected by Ulysses, so far the only spacecraft able to measure in situ plasma velocity, density, and magnetic field outside the ecliptic plane. The high-latitude fast solar wind is a quintessential example of expanding nonrelativistic astrophysical plasma, which we choose to investigate during a period of low solar activity to minimize the effects of nonstationarity and inhomogeneity, potentially occurring in the observed fields. Although based on the computation of the structure functions of the Elsässer variables, the third-order fitting procedure we employ does not require the existence of linear scaling regimes (*56*), as opposed to classical asymptotic laws (*37*).

When computed locally in time—whether from numerical simulations or, as in this case, from observations within relatively short temporal windows—*F*(*r*) is expected to be a fluctuating quantity. Our approach provides also the scale-dependent energy injection and dissipation rates within the range of scales accessible for the given observational samples (*54*, *56*), hereafter indicated as $\epsilon_{\text{LOC}}$ , which is critical for an accurate physical interpretation of the energy fluxes estimated from observations. From *F*(*r*) one retrieves the turbulent fluxes toward or from scales smaller [ $F_{\text{small}}=F\left(\ell \right)$ ] and larger [ $F_{\text{Large}}=-F\left(L\right)$ ] than the range of scales considered. The extent of this range depends on the separation between the smallest spatial scale resolved ( ℓ ), set in this case by the instruments resolution on board of Ulysses, and the size of the solar wind samples analyzed ( L ). Positive/negative values of $F_{\text{Large}}$ imply energy transferred to/from scales larger than the domain size, whereas positive/negative values of $F_{\text{small}}$ imply energy transferred to/from smaller unresolved scales. A dual cascade caused by energy injections occurring within the accessible range of scales is consistent with values of the fluxes $F_{\text{Large},\text{small}}>0$.

**Fig. 1.**
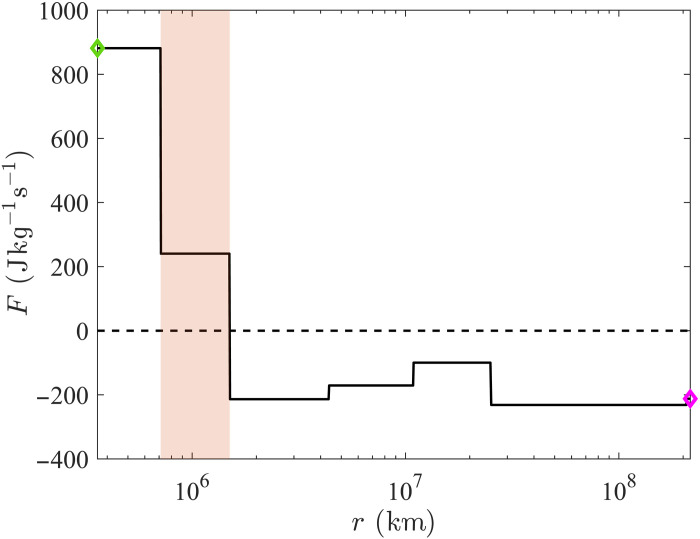
Local-in-scale turbulent energy flux. The black line is the scale-to-scale energy flux *F*(*r*) characterizing solar wind observed by the spacecraft Ulysses during the days from 115 to 126 of year 1996. The red shaded area denotes the dominant energy injection scales; the magenta/green diamond marks the rate at which total energy is transferred to scales larger/smaller than the largest/smallest scale available for the solar wind sample analyzed, defined as $F_{\text{Large}}=-F\left(L\right)$ and $F_{\text{small}}=F\left(\ell \right)$ , respectively, with $L≃2.16\times 10^{8}\text{km}$ and $\ell ≃3.6\times 10^{5}$ km.

Figure 1 reports the scale-to-scale energy flux *F*(*r*) computed over a 11-day window of the Ulysses time series (days interval 115 to 126 of year 1996), a sample size validated in previous investigations (*32*, *57*). The spatial scales on the horizontal axes obtain from the conversion of the temporal increments τ built on the spacecraft’s time series, assuming the Taylor’s frozen hypothesis as valid (*58*). Thus, r=−〈U〉τ , where 〈U〉 is the average solar wind speed, fairly constant in the dataset considered here (~750 km/s). The green-shaded area in Fig. 1 indicates the dominant energy injection scales (found at ~10^6^ km) in the solar wind sample analyzed. For scales smaller than *O*(10^6^)km, the energy flux is positive and relatively constant, although over a short range, limited by the instruments resolution. For scales larger than *O*(10^6^)km, the flux is instead negative and nearly constant over a range of more than two decades, indicating a robust inverse energy transfer, which demonstrates the existence of a dual energy cascade in the fast solar wind. Here, $F_{\text{Large}}≃200{\text{Jkg}}^{-1}s^{-1}$ and $F_{\text{small}}≃900{\text{Jkg}}^{-1}s^{-1}$ (magenta and green diamonds in Fig. 1, respectively) indicate that the total energy keeps cascading to scales larger than ~1/3 of the size of the solar wind sample analyzed ( $L≃2.16\times 10^{8}\text{km}$ , corresponding to ~3 days), the largest scale considered in this analysis, and smaller than the smallest resolved scale ( $\ell ≃3.6\times 10^{5}\text{km}$ , corresponding to 8 min, the sampling time of the Ulysses plasma instrument).

### Dominant energy injection scales

**Fig. 2.**
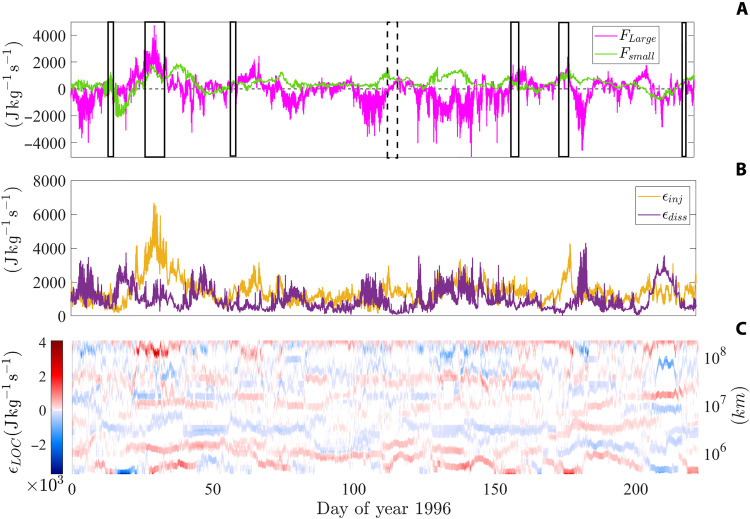
Temporal evolution of turbulent fluxes, injection and dissipation rates computed on the Ulysses time series. (**A**) Total energy fluxes to scales larger/smaller than the largest/smallest available scale, $F_{\text{Large}}$ and $F_{\text{small}}$ , respectively. (**B**) Energy injection and dissipation rates, $\epsilon_{\text{inj}}$ and $\epsilon_{\text{diss}}$ , respectively, computed in 11-day windows covering the fast solar wind database analyzed. (**C**) By-scale energy injection and dissipation rates, corresponding to positive and negative values of $\epsilon_{\text{LOC}}\left(r\right)$ , respectively. The boxes highlight solar wind segments characterized by bidirectional energy transfers, compatible with the dual cascade scenario. The dashed box denotes the interval selected for the analysis reported in Fig. 1.

**Fig. 3.**
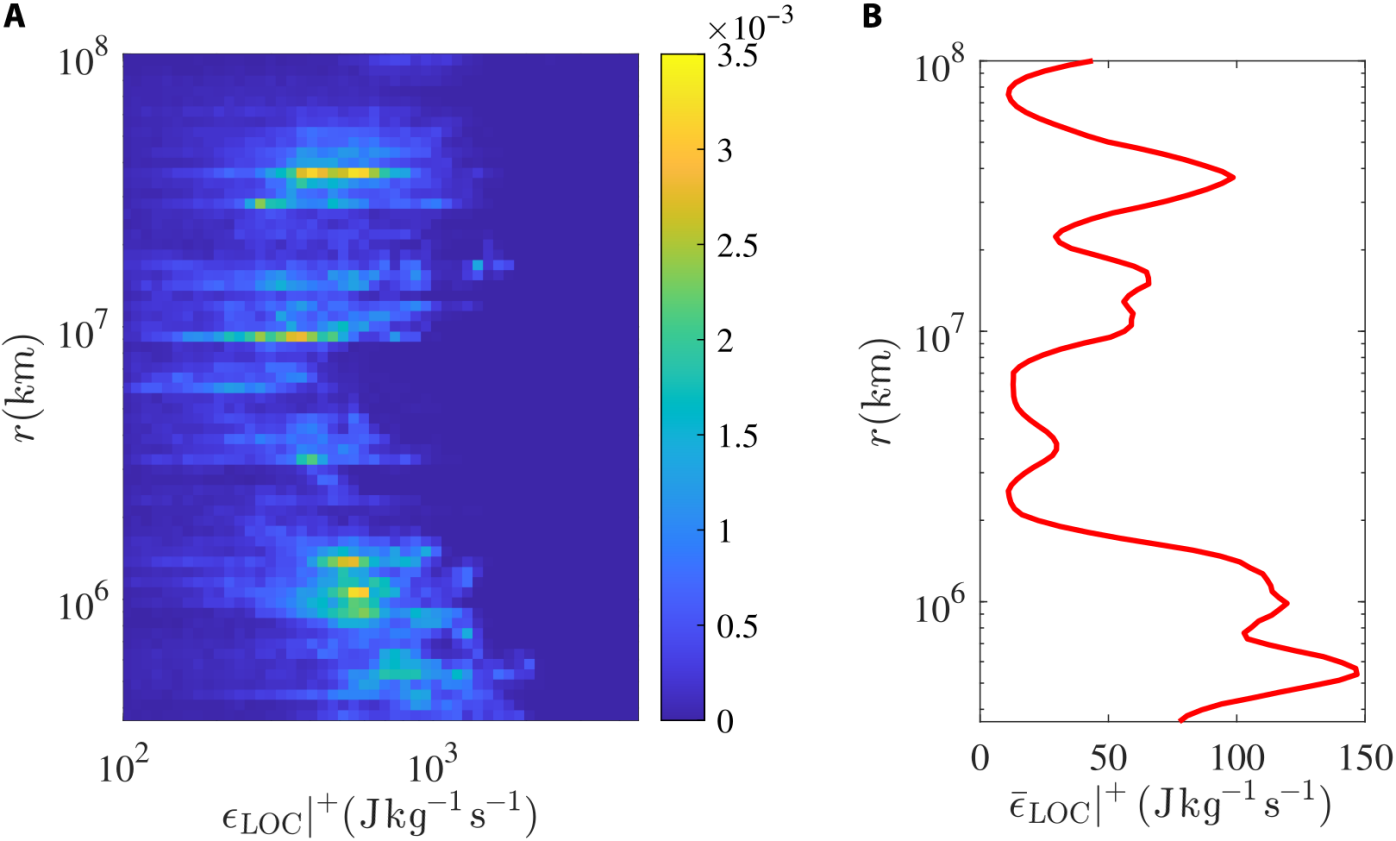
Statistics of the energy injections. (**A**) Joint probability distribution of the total energy injection rate, ${\epsilon_{\text{LOC}}\left(r\right)|}^{+}$ , versus the scale (*r*). (**B**) Mean values of the by-scale total energy injection rate, ${\bar{\epsilon }{\left(r\right)}_{\text{LOC}}\left(r\right)|}^{+}$ . This quantity is obtained by horizontally averaging ${\epsilon_{\text{LOC}}\left(r\right)|}^{+}$ in (A). Statistics are computed over the whole Ulysses database analyzed, thus cumulated through the time domain indicated in Fig. 2.

We performed systematically the analysis leading to Fig. 1 on a 11-day window running through ~7 months of velocity and magnetic field data collected by Ulysses in the high-latitude fast solar wind, covering a large portion of the year 1996. While only a fraction of the windows analyzed exhibit clear constant positive and negative turbulent flux regimes—as expected, given the fluctuating nature of *F*(*r*)—the fact that $F_{\text{Large}}$ and $F_{\text{small}}$ are always well defined allowed us to compute temporal statistics of the bidirectional energy flux over a large number of windows throughout the Ulysses database. Figure 2A displays $F_{\text{Large}}$ and $F_{\text{small}}$ , whereas Fig. 2C illustrates the local-in-scale total energy injection or dissipation rate, $\epsilon_{\text{LOC}}\left(r\right)$ , depending on whether its sign is positive or negative, respectively. At each time, scale integrations of $\epsilon_{\text{LOC}}\left(r\right)$ over its positive and negative values, separately, provide respectively the energy injected in ( $\epsilon_{\text{inj}}$ ) or removed from ( $\epsilon_{\text{diss}}$ ) the accessible range of scales for the solar wind samples analyzed, shown in Fig. 2B. Eventually, the scalogram in Fig. 2C allows to identify the scales at which the energy is mostly injected or removed from each solar wind sample window, again depending on the sign of $\epsilon_{\text{LOC}}\left(r\right)$ . Although the signal is highly intermittent, strong positive peaks of $\epsilon_{\text{LOC}}\left(r\right)$ dominate the energy injection, persisting throughout the time series. Robust peaks are found also in the joint probability distribution shown in Fig. 3A, where statistics of the positive values of $\epsilon_{\text{LOC}}\left(r\right)$ , hereafter ${\epsilon_{\text{LOC}}\left(r\right)|}^{+}$ , are integrated over the entire temporal axes of Fig. 2. Last, averaging over each horizontal bin of the distribution function in Fig. 3A provides the by-scale mean energy injection rate ${{\bar{\epsilon }}_{\text{LOC}}\left(r\right)|}^{+}$ characterizing the ~7 months of solar wind analyzed, shown in Fig. 3B. Consistent with the color patterns emerging in the scalogram of Fig. 2C, the largest peak of ${{\bar{\epsilon }}_{\text{LOC}}\left(r\right)|}^{+}$ occurs between ~3 × 10^5^ km and ~2 × 10^6^ km. These scales are compatible with the size of the typical solar coronal loops detected during solar activity minima at heliolatitudes within [20°:60°] North (that include those explored by Ulysses in the period under study), ranging from about 100 to 800 Mm (*59*). Less intense and/or frequent energy injections occur also at scales between ~10^7^ and ~10^8^ km, which might instead be consistent with the presence of velocity shears in the high-latitude Ulysses time series, as reported in (*60*).

### Equipartition of the turbulent flux

**Fig. 4.**
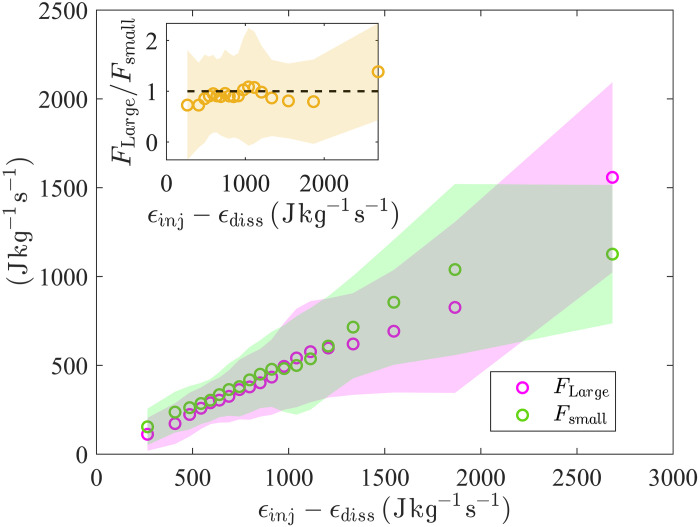
Turbulent flux equipartition. Dependence of large- (magenta) and small-scale (green) energy fluxes on the net energy injection rate. $F_{\text{Large}}$ and $F_{\text{small}}$ are averaged in bins of $\epsilon_{\text{inj}}-\epsilon_{\text{diss}}$ , the associated SDs are plotted as shades. The flux ratio in the insert ( ≃1 within errors) demonstrates that equipartition is achieved statistically. Statistics here are computed considering only the fast solar wind windows characterized by bidirectional energy transfers.

The solar wind windows characterized by the occurrence of simultaneous upscale and downscale energy transfer, identified by the condition $F_{\text{Large},\text{small}}>0$ , and where the net energy injection is positive, corresponding to $\epsilon_{\text{inj}}-\epsilon_{\text{diss}}>0$ , such as the solar wind segments within the boxes in Fig. 2A, are compatible with the dual energy cascade scenario. The bidirectional energy transfer as detected through the above conditions is observed in about 40% of the windows analyzed, as it can be assessed from Fig. 2. For this subset of cases, plotting the average values of $F_{\text{Large}}$ and $F_{\text{small}}$ computed in statistical bins of the net energy injection $\left(\epsilon_{\text{inj}}-\epsilon_{\text{diss}}\right)$ leads to the important result shown in Fig. 4: not only both upscale and downscale total energy fluxes, $F_{\text{Large}}$ and $F_{\text{small}}$ , respectively, are linearly proportional to the net energy injected in the high-latitude fast solar wind windows analyzed, but there is also—on average—a clear equipartition of the turbulent flux toward scales larger than the scale of the domain flow and smaller than the smallest resolved scale, as demonstrated by the ratio $F_{\text{Large}}/F_{\text{small}}$ , shown in the insert, being always close to unity. The shaded areas are the standard deviations associated to the estimates of $F_{\text{Large}}$ , $F_{\text{small}}$_,_ and their ratio and are computed in each statistical bin on the horizontal axis. It is important to emphasize that the methodology used here was key to obtaining this result. Our third-order approach is applicable in the presence of energy injections occurring within the range of scale in which turbulent cascades are expected to develop and does not require the existence of linear scaling regimes, as previously noted. In the present analysis, the estimates of the scale-to-scale energy flux, *F*(*r*), can also be considered as local in time—the fluxes being computed in relatively short temporal windows. When averaged over long periods (or across many 11-day windows), the statistics of $F_{\text{Large}}$ and $F_{\text{small}}$ , whose local values fluctuate as the plasma evolves in time (as is typical in turbulent flows), are expected to converge to the global large- and small-scale total energy fluxes. This analysis was also made possible thanks to the Ulysses observations, which provided solar wind signals under generally stationary conditions of the solar input (i.e., during low solar activity), far from the disturbances characterizing the ecliptic plane.

## DISCUSSION

In a complex multiscale turbulent system as encountered in many astrophysical environments, energy is possibly injected at several scales. Given nonlinear couplings due to the several forces that are present, this energy can be transferred to both larger and smaller scales through mode coupling, but whether it does so in both directions and in each case with a constant flux is a more difficult question that has only been tackled recently. Observed in the most disparate frameworks, at vastly different scales, from geophysical fluids (*54*, *61*–*63*) to quantum fluids (*64*), kinetic plasmas (*65*), and now in the space, bidirectional energy transfers increasingly emerge in the study of turbulent systems as a universal feature, characteristic of certain regimes of the complex dynamics of fluids and plasmas at high Reynolds numbers (*66*, *67*).

Together with its relevance for the advancement of fundamental plasma and turbulence theories, the rigorous characterization of the sign of the turbulent energy flux in astrophysical plasmas is important to shed light on such major open questions as the solar wind heating (*18*) and the role of turbulence in star formation (*8*). Progress in understanding how small-scale dissipation is accomplished in noncollisional astrophysical plasmas shall depend as well on our capability to assess the fraction of energy that cascades from the magnetohydrodynamic regime down to the kinetic regime or else undergoes an inverse transfer feeding large-scale structures and potentially also waves. Features of the magnetohydrodynamic energy cascade may play a critical role in determining the energy budget of even the smaller kinetic scales, where equilibria could develop as a result of cross-scale energy couplings and conversion due to mechanisms such as magnetic reconnection, triggering local turbulence (*16*, *65*). All that makes the investigation of the turbulent energy transfer across plasma regimes a priority task of present and future space missions (*51*, *68*–*71*).

Following the theory in (*30*, *31*), previous observations of negative linear scaling regimes of the third-order structure functions of the Elsässer variables suggested the possibility of an inverse energy transfer in solar wind (*32*, *43*, *52*). On the other hand, only the knowledge of the local-in-scale energy flux [*F*(*r*)] and the identification of the dominant forcing scales allow to establish unambiguously the existence of dual energy cascades in astrophysical plasma turbulence.

In this study, we have proposed and demonstrated that a different investigation technique (*54*, *56*) allows the discovery of bidirectional energy transfers in the fast solar wind, simultaneously to large and to small scales, leading statistically to equipartition of the turbulent flux. The observed forward and inverse energy transfers can be characterized by regimes with nearly constant positive and negative scale-to-scale fluxes, which is consistent with the definition of cascades in turbulence (*72*). The analysis presented suggests that the most powerful energy injections throughout the high-latitude solar wind under study occur at scales compatible with typical coronal loop sizes (*59*). This reinforces the conclusions of recent investigations performed using remote sensing observations by Solar Orbiter (*68*), providing evidence of the onset of fully developed turbulence at the level of the solar corona (*73*). Our analysis shows that energy injections can occur with variable intensity over a broad range of scales in solar wind. In addition to large solar coronal loops (*74*), various smaller-scale structures and phenomena may also play an important role in driving turbulent dynamics in the interplanetary plasma. These include granules in the chromosphere (*75*), solar wind shear (*27*, *60*), magnetic switchbacks (*76*–*78*), and magnetic reconnection events (*16*, *65*), all of which are potential triggers of turbulent energy transfer and magnetic-to-kinetic energy conversion. Collected far from the Sun and unaffected by the strong shears, compressions, and inhomogeneities that develop in the ecliptic plane, the Ulysses solar wind samples analyzed here allowed us to explore features of the turbulent energy flux that may characterize expanding nonrelativistic stellar plasmas in the universe. Along with magnetic fields (*6*), turbulence contributes to the pressure of the interstellar medium, supporting molecular clouds in galaxies by counteracting their self-gravity (*7*, *8*). Once a certain number of stars have formed, stellar feedback becomes significant and may disrupt the cloud. However, what regulates star formation within molecular clouds—whether magnetic fields, large-scale turbulent motions driven by galactic dynamics, or internal stellar feedback—remains a subject of debate. The joint action of gravity and turbulence is undoubtedly central to structuring both the interstellar and intergalactic media (*79*). Yet, the precise mechanisms through which this occurs remain among the major open questions in astrophysics, partly due to the complexity of cosmic turbulence, which involves plasma dynamics and permeates all thermal phases. The results presented here suggest that a non-negligible fraction of the energy in stellar winds may be transferred from small to large scales via plasma turbulence. Although we are unable to provide a quantitative estimate of the efficiency of this mechanism in astrophysical plasmas beyond the solar wind, our study indicates an alternative pathway by which turbulence can transport energy from stellar to larger scales, potentially contributing to star formation processes. This insight could be important not only from a fundamental perspective, to support the development of more comprehensive theoretical models, but also for improving subgrid-scale parameterizations in full galactic to star scale simulations.

## MATERIALS AND METHODS

### Spacecraft Ulysses data

We analyzed continuous time series of plasma velocity, density (of protons and alpha particles), and magnetic field collected by the spacecraft Ulysses in the high-latitude fast solar wind during a period of low solar activity. These correspond to roughly ~7 months of in situ observations during which spacraft’s distance from the Sun and helilatitude exhibited small and slow variations: from 3 to 4 au and from 55° to 30°, respectively. For this study, Ulysses data were first resampled at a uniform 8-min interval and then used to compute the Elsässer variables. Solar wind is a multiscale framework characterized by high spatial and temporal variability of all its physical fields. The dataset to analyze in this study was therefore chosen to minimize the dependence of turbulent dynamics on solar activity and orbital parameters of the spacecraft, avoiding the complexity of the interplanetary plasma in the proximity of the ecliptic plane and close to the Sun—caused by strong shears between fast and slow solar wind streams, compressions, and inhomogeneities. The Sun’s poles are covered by coronal holes; thus, they are characterized by the absence of active regions. The high-latitude solar wind is, as a consequence, the most stationary, especially during solar minima. That makes the fast solar wind considered in this study the most suitable to perform high-order statistics under the assumptions of homogeneity, isotropy, and incompressibility. Ulysses was launched in 1990 and, as of today, is the only spacecraft to have explored the high-latitude heliosphere (*80*). Eleven-day windows of the Ulysses time series contain from ~10 to ~10^2^ correlation lengths of the Elsässer fields, representing a good compromise between ensuring statistical convergence, preserving stationarity and mitigating potential effects of solar wind expansion (*32*, *57*).

### Forcing-resolving third-order analysis

The analysis presented is conducted through the implementation of the forcing-scale resolving third-order approach proposed by Xie and Bühler (*56*), briefly described here. Under the assumption of homogeneity, isotropy, and incompressibility, an expression for the third-order structure function of the Elsässer variables, $V\left(r\right)=\langle {|\Delta z^{+}\left(r\right)|}^{2}\Delta z_{\|}^{-}\left(r\right)\rangle +\langle {|\Delta z^{-}\left(r\right)|}^{2}\Delta z_{\|}^{+}\left(r\right)\rangle$ , is obtained

$$\begin{array}{c} V\left(r\right)=\frac{4}{3}F_{\text{up}}-4\epsilon \frac{r_{f}^{3}}{r^{2}}\left[\text{sin}\left(r/r_{f}\right)-k_{f}r\text{cos}\left(r/r_{f}\right)\right] \end{array}$$ (2)

As it was shown in the context of the ocean (*54*), this model is able to capture a dual cascade scenario, where energy is injected at scale $r_{f}$ with a rate ϵ , and the upscale and downscale energy fluxes at scales larger and smaller than the forcing scale are $F_{\text{up}}$ and $F_{\text{down}}=\epsilon -F_{\text{up}}$ , respectively. Here, ϵ>0 (or <0 ) means energy injection (or dissipation). Following the mathematical theory developed in (*56*), the relation obtained between the scale-to-scale energy flux *F*(*r*) and the parameters in Eq. 2 is

$$F\left(r\right)=\epsilon H\left(r_{f}-r\right)-F_{\text{up}}$$ (3)

where *H* is the Heaviside function and a negative (positive) value of *F* corresponds to an energy flux towards larger (or smaller) scales. This approach requires to fit the third-order structure function computed from observational data with a fitting function that is a linear combination of Eq. 2

$$\begin{array}{c} V\left(r\right)=\frac{4}{3}{\tilde{F}}_{\text{up}}-\sum_{i=1}^{N}\frac{4\epsilon_{i}r_{\mathrm{fi}}^{3}}{r^{2}}\left[\text{sin}\left(r/r_{\text{fi}}\right)-k_{\text{fi}}r\text{cos}\left(r/r_{\text{fi}}\right)\right] \end{array}$$ (4)

where $\epsilon_{i}$ is the energy injection rate at the scale $r_{\text{fi}}$ and the total energy injection rate within the accessible range of scales is $\epsilon =\sum_{i=1}^{N}\epsilon_{i}$ . For a given observational data sample, ${\tilde{F}}_{\text{up}}$ is the energy flux through the largest detected scale, whereas the energy flux through the smallest detected scale (normally set by the instruments’ resolutions) is ${\tilde{F}}_{\text{down}}=\epsilon -{\tilde{F}}_{\text{up}}$ . The scale-to-scale energy flux within the accessible range of scales is eventually

$$F\left(r\right)=\sum_{i=1}^{N}\epsilon_{i}H\left(r_{\text{fi}}-r\right)-{\tilde{F}}_{\text{up}}$$ (5)

The relation (*4*) with prescribed $r_{fi}$ is used to perform fits of the third-order structure function built on the solar wind data [*V*(*r*)] to obtain the energy injection rates relative to the different scales ( $\epsilon_{i}$ ). Previously, this linear procedure was successfully implemented on observations of the velocity field in the ocean (*54*). The methodology can induce amplifications of the small-scale errors when $\epsilon_{i}$ is not sign definite. For this reason, a nonlinear fitting procedure of the structure function [V(r)] is proposed, where both energy injection scale ( $r_{fi}$ ) and energy injection rate ( $\epsilon_{i}$ ) are treated as fitting parameters [see the Supplementary materials in (*54*)]. Specifically, to avoid fast oscillatory modes that may amplify the errors, the ranges of $k_{\text{fi}}$ are constrained based on the following strategy: an equal partition of the scale space in the log coordinates is made into *N* intervals, and the $r_{\text{fi}}$ ranges are restricted in the *i*th interval; thus, most of the forcing scales pairs cannot be too close. The nonlinear curve fitting function “lsqcurvefit” in MATLAB is lastly used to retrieve the parameters of interest from the observational data.

The analysis presented here was entirely conducted using the Elsässer fields, so that the physical quantities indicated as $\epsilon_{\text{inj},\text{diss},\text{LOC}}$ and $F\left(r\right),F_{\text{Large}},F_{\text{small}}$ , appearing in the manuscript, are injection/dissipation rates and fluxes, respectively, of the total energy, defined as the sum of the solar wind’s kinetic and magnetic energy.
